# Time in range: a new parameter to evaluate blood glucose control in patients with diabetes

**DOI:** 10.1186/s13098-020-00529-z

**Published:** 2020-03-16

**Authors:** Monica Andrade Lima Gabbay, Melanie Rodacki, Luis Eduardo Calliari, Andre Gustavo Daher Vianna, Marcio Krakauer, Mauro Scharf Pinto, Janice Sepúlveda Reis, Marcia Puñales, Leonardo Garcia Miranda, Ana Claudia Ramalho, Denise Reis Franco, Hermelinda Pedrosa Cordeiro Pedrosa

**Affiliations:** 1 Diabetes Centre-UNIFESP, Federal University of São Paulo, São Paulo, Brazil; 2 Nutrology and Diabetes Section, Internal Medicine Department Federal University of Rio de Janeiro–UFRJ, Rio de Janeiro, Brazil; 3 Pediatric Endocrinology Unit, Pediatric Department, Santa Casa de São Paulo School of Medical Sciences, São Paulo, Brazil; 4 Curitiba Diabetes Center, Department of Endocrine Diseases, Hospital Nossa Senhora das Graças, Curitiba, Brazil; 5 ABC Medical School, São Paulo, Brazil; 6 Santa Casa of Belo Horizonte, Belo Horizonte, Brazil; 7 Institute of Child with Diabetes, Conceição Children Hospital, Conceição Hospitalar Group, Porto Alegre, Brazil; 8 Unit of Endocrinology and Research Center, Regional Hospital of Taguatinga, Secretariat of Health of the Federal District, Brasilia, Brazil; 9 Department of Endocrinology Federal University of Bahia, Bahia, Brazil; 10 CPCLIN/DASA Clinical Research Center, São Paulo, Brazil; 11 Head of Diabetes Research Centre of Endocrinology Unit, Secretariat of Health of the Federal District, Brasilia, Brazil

**Keywords:** Time in range, Glycated hemoglobin, Continuous glucose monitoring, Hypoglycemia

## Abstract

The International Consensus in Time in Range (TIR) was recently released and defined the concept of the time spent in the target range between 70 and 180 mg/dL while reducing time in hypoglycemia, for patients using Continuous Glucose Monitoring (CGM). TIR was validated as an outcome measures for clinical Trials complementing other components of glycemic control like Blood glucose and HbA1c. The challenge is to implement this practice more widely in countries with a limited health public and private budget as it occurs in Brazil. Could CGM be used intermittently? Could self-monitoring blood glucose obtained at different times of the day, with the amount of data high enough be used? More studies should be done, especially cost-effective studies to help understand the possibility of having sensors and include TIR evaluation in clinical practice nationwide.

## Background

The International Consensus in Time in Range (IC-TIR) [1] was recently released and the purpose of this manuscript is to critically discuss TIR and to offer diabetologists and endocrinologists concise and meaningful information. This technical review commentary expresses Brazilian experts’ opinion on this interesting metric obtained through continuous glucose monitoring (CGM) and represents a demand requested by the Brazilian Diabetes Society to translate the IC-TIR to the national practice.

## Main text

The International Consensus in Time in Range (IC-TIR) [1] was recently released and the purpose of this manuscript is to critically discuss TIR and to offer diabetologists and endocrinologists concise and meaningful information. This technical review commentary expresses Brazilian experts’ opinion on this interesting metric obtained through continuous glucose monitoring (CGM) and represents a demand requested by the Brazilian Diabetes Society to translate the IC-TIR to the national practice.

The benefits of achieving normal or near-normal blood glucose levels are well known since the Diabetes Control and Complications Trial (DCCT) [2]. Hemoglobin A1c test (HbA1c) has been used as a gold standard of glycemic control since DCCT, while the self- monitoring blood glucose (SMBG) has been a cornerstone of diabetes care to verify glucose variability (GV) on daily basis [3].

HbA1c reflects blood glucose concentrations over three to four months and is the only parameter of glycemic control that has strongly been associated with chronic diabetic vascular complications. “However, HbA1c may be influenced by several conditions that affect the survival of red blood cell (RBC) independent of glycemia, but also by glycation rates, uremia, pregnancy, smoking, and ethnicity. Higher HbA1C values have been described in minorities, mainly African Americans, for example. All these factors affect the interpersonal relationships between HbA1c and mean glucose. The degree of such impact is currently immeasurable and frequently not fully appreciated. When these other conditions influencing HbA1c levels are considered, it becomes clear that the relationship between HbA1c and complications may not be the same as the relationship between mean blood glucose and complications [4]”.

HbA1c does not distinguish individuals with similar average glycemia but with pronounced differences in hypoglycemic events and/or hyperglycemic excursions [4, 5]. SMBG provides a “snapshot” of the glucose values and it is used both to titrate prandial insulin doses and to define correction bolus, but does not detect fluctuations that might occur between each capillary glucose test unless testing is done consecutively over short periods.

CGM provides a continuous measurement of the interstitial glucose over time and offers the opportunity to detect glucose variations, hypoglycemic events, and time in range (TIR) [4]. Both real-time CGM (rtCGM) or intermittent scan CGM (isCGM) are currently available [6]. The main benefit of CGM is observed in high-risk patients with frequent or severe hypoglycemia, and those with impaired awareness of hypoglycemia. CGM can be effectively used in patients either in multiple daily injections (MDI) treatment or in those with continuous subcutaneous insulin infusion (CSII).

In 2017, an International Consensus on the Use of the Continuous Glucose Monitoring [7] standardized the use of CGM and recommended the analysis together with HbA1c to promote therapy adjustments in both type 1 (T1DM) and type 2 (T2DM) diabetes mellitus, especially for patients with frequent hypoglycemia. The consensus also recommended that all patients should be trained in how to access, interpret, and answer questions regarding their glycemic control in the available devices and tools. Definitions of the minimum requirements for CGM performance, such as meeting ISO (International Organization for Standardization) standards, the relationship of dependence of CGM calibration with glucometers, and an acceptable mean absolute relative difference (MARD) were provided.

The consensus also considered hypoglycemia definitions as clinical trial standardization and divided them into levels 1, 2, and 3, based on the joint position statement of the American Diabetes Association (ADA) and the European Association for the Study of Diabetes (EASD) following the recommendations of the International Hypoglycaemia Study Group (IHSG) [8]. This Time Bellow Range (TBR) was divided into Level 1 (between 54 and 70 mg/dL) has minor importance in clinical studies. Level 2 (below 54 mg/dL) has major clinical significance and must be reported. Level 3 hypoglycemia is considered severe, whenever assistance by third parties is necessary, without a specific value of blood glucose. Hypoglycemic event is considered if lasting at least 15 min. The cessation of a hypoglycemic episode should be considered 15 min after the glycemia reaches values outside that range. Hyperglycemic exposure is expressed as the percentage of time with glucose values > 180 mg/dL. Hyperglycemia (Time Above Range or TAR) is also divided into three levels level 1 (alert level, > 180 mg/dL to < 250 mg/dL), level 2 (clinically significant, > 250 mg/dL) and level 3 (clinical diagnosis: ketoacidosis or hyperosmolar hyperglycemic state). Splitting the time in hypo and hyperglycemia into three levels allows a more assertive assessment of severity and the most appropriate response.

“The recommended amount of data is 100% in at least 10 days or 70% of captured data in at least 14 days of CGM. This metric, based on the ADAG study has been called “estimated HbA1c” or just “eA1c”, and is present in some reports of CGM devices. However, the use of this term started to generate confusions when values of “real A1c”, measured in the blood, were not similar to “eA1c”, estimated by CGM data. Health care professionals and patients had difficulties in interpreting these differences, and the FDA (Food and Drug Administration) suggested that the name should be changed [9]. Based on these arguments, Bergenstal et al. used data coming from novel CGM studies associated to the previous ADAG results to develop a new index, the glucose management indicator (GMI) [10]. The FDA supported the use of the term GMI, and probably it will be used in the reports of different CGM devices from now on [11].”

Finally, the consensus defined the concept of the time spent in the target range, or simply “time in range” and standardizes the use of the primary glucose range between 70 and 180 mg/dL. Occasionally, glucose values between 70 and 140 mg/dL can be used as a secondary range, especially for regulatory issues and comparability studies. Before 2017 consensus, time in target ranges were reported in various ways, and it was impossible to compare one study with the others. The consensus agreement finished a discussion about what would be the best metric to be used. In 2019, the IC-TIR recommended clinical targets for CGM data for T1DM and T2DM, at-risk or “frail” patients with diabetes and established a specific recommendation for pregnancy. Moreover, percentages of time in hypoglycemia and hyperglycemia were also a matter of the IC-TIR consensus (Table 1).

**Table 1:** Guidance on target for assessment of glycemic control in patients with diabetes

	TIR	Time in hypoglycemia	Time in hyperglycemia
T1DM and T2DM	> 70% (70–180 mg/dL)	< 4% below 70 mg/dL < 1% below 54 mg/dL	< 25%
T1DM and T2DM “fragile”	> 50% (70–180 mg/dL)	< 1% below 70 mg/dL	> 90% below 250 mg/dL
T1DM pregnancy	> 70% (63–140 mg/dL)	< 4% below 63 mg/dL	< 25% above 140 mg/dL
Gestational DM and T2DM pregnancy^a^	> 85–90% (63–140 mg/dL)	< 4% below 63 mg/dL	< 10% above 140 mg/dL

^a^Gestational DM and T2DM pregnancy: there are no specific recommendations for these conditions given the limited evidence but that it is expected that it would be significantly higher than in type 1 diabetes pregnancy

Evaluation of CGM metrics is essential to motivate, educate and teach patients with diabetes in clinical practice. The aim is to reduce the time spent in hypoglycemia (glucose levels < 70 mg/dL) to less than 1 h/day and time below 54 mg/dL to less than 15 min/day, equivalent to < 4% and < 1%, respectively as the standard goal. Indeed, targets must be individualized and meet personal needs and circumstances [1, 7, 12].

The article published by ADA/EASD, entitled “Improving the Clinical Value and Utility of CGM Systems: Issues and Recommendations” [13] motivated an editorial by Riddle, Gerstein, and Cefalu highlighting thought-provoking points about CGM [14]. They supported the definition of several terms and ways of reporting a standardized CGM and the classification and report of hypoglycemia. Additionally, they stressed the importance of this standardization for a paradigm shift in regulatory affairs. Another important aspect to be pointed out is that monitoring the time in range can also offers an opportunity for people with diabetes to improve the management of their diabetes.

In a recent publications IQVIA developed the CORE Diabetes Model, that simulates clinical outcomes and costs for cohorts of patients with diabetes. The authors demonstrated that improvement in time in range to 80% and reducing hypoglycemic events by up to 40% can, conservatively, lead to a reduction in costs of $6.7–9.7 billion over 10 years in USA. This publication, based on recent studies by Beck et col [15] and Vigersky et col [16], also predicts that an increase in TIR reduces the cumulative incidence of developing complications such as myocardial infarction, end stage renal disease, severe vision loss and amputation [17].

Some questions are still not answered such as: who should use CGM and when, and who should pay for it? It is described that there might be different definitions for specific ethnic groups and there are still open doors for a better understanding concerning CGM, cardiovascular risk and GV.

Following the publication of the CGM consensus in 2017, new data were published on the importance and usefulness of TIR. To validate TIR as an outcome measure for clinical trials, Beck et al. [15], reanalyzed the dataset of DCCT study [2]. Using DCCT’s capillary measurements, the authors searched for associations between TIR and the development or progression of microalbuminuria or retinopathy. All 1440 DCCT participants measured a 7-point glucose profile from fingerstick samples for 1 day every 3 months. In total, blood glucose (BG) testing data were available for 32,528 quarterly data collections, with the 7-point profile complete for 24,892. The correlation between mean TIR and HbA1c was − 0,7913. TIR was higher in the intensively treated group than in the conventionally treated group (52 vs. 31%). Although the information coming from BG measurements was not so complete as it would have been with CGM, the massive amount of blood glucose tests could be used as a good representation of the glucose profile of DCCT population. Pitfalls are that the 7-point profile represents only daytime measures and that this study was performed only in patients with T1DM. These results do not apply for patients with T2DM, although we can speculate that in T2DM patients it is likely that the same associations would be present. That would possibly imply a further correlation with the UKPDS [18] or any other robust data with mainly T2DM.

Lu et al. have investigated the relationship between retinopathy and TIR evaluated through CGM in patients with T2DM. The prevalence of retinopathy was higher in patients with lower TIR. Moreover, patients with more advanced retinopathy had less TIR and higher measures of glucose variability [19]. Similarly, Mayeda et al. have shown an association between TIR evaluated through CGM and symptoms of peripheral neuropathy in individuals with T2DM [20]. However, these studies evaluated only short term CGM in patients with long-standing disease and did not include TIR data during the course of the disease.

Recently, a commentary by Hirsh et al. has drawn attention to the fact that TIR was relatively low in the DCCT data (52% vs. 31%, intensive vs. conventional treatment, respectively) [21]. The difference in TIR between the groups that developed retinopathy and others was 12%, while for those with or without microalbuminuria a difference of only 10% was reported. The difference in TIR between those that developed eye or kidney disease and others was a decreasing of approximately 2.5 h per day in the range, which emphasized the critical role of TIR measurement. The authors concluded that TIR is strongly associated with the risk of microvascular complications, and therefore could be used as another endpoint for clinical investigations.

“Recent studies have demonstrated the relationship between TIR and complications in T2D patients. Lu et al. initially investigated the association between the TIR, assessed by CGM, and diabetic retinopathy (DR) in 3262 patients. Patients with more advanced DR had significantly less TIR and higher measures of GV (p < 0.01) with significant associations between TIR and all stages of DR [19]. Then, the same group analyzed carotid intima-media thickness (CIMT) of 2215 T2D patients and found a correlation between TIR and macrovascular disease. Those with abnormal CIMT had significantly lower TIR (p < 0.001) and each 10% increase in TIR was associated with 6,4% lower risk of abnormal CIMT [22]. It should be noted that all subjects in these studies underwent 3 days of CGM, while previous ones demonstrated that increasing the number of days of CGM improved the correlation of CGM data with the glucose metrics over 3 months, and that 12–15 days of CGM may be needed to optimally evaluate glycemic control”.

Articles that report paired HbA1c and TIR metrics or HbA1c and frequent self-monitoring of blood glucose points out TIR as a new tool for determining the outcome of clinical studies. Vigersky and McMahon [16] analyzed 18 studies including 2577 T1DM and T2DM subjects and found a strong relationship between TIR and HbA1c (R = − 0,84; R^2^ = 0,71). It was demonstrated that for every 10% change in TIR, there was a 0.8% change in HbA1c. TIR and HbA1c are not efficient for estimating the time in hypoglycemia (time below range), so composite metrics (TIR + time below range) are suggested to be complementary to HbA1c. A limitation of the study is that most of the subjects were white and non-Hispanic. Since the relationship between HbA1c and average glucose differs by race/ethnicity, the findings of this study may be inaccurate for non-Caucasians.

Additionally, TIR could be a useful metric along HbA1c to assess glycemic control in children. Petersson et al. [23] evaluated 133 children and adolescents from Sweden that used rt-CGM or isCGM and demonstrated a non-linear correlation between TIR (70–140 mg/dL) and HbA1c for 60 days (R^2^ = 0,69).

The opinion of the SBD experts invited group, the strength of the study is that the authors collected sensor data from CGM only when the sensor has been used for more than 80% of the time. They found a strong relationship between TIR, time above range and HbA1c, but only a modest association with hypoglycemia. The weakness is that they used a stricter range for calculation of TIR (70–140 mg/dL). Although these data were obtained from a pediatric population, the study partially validated the concept of TIR in this population, since in previous studies a linear relation between HbA1c and TIR has been shown in subjects with T1DM and T2DM.

“In a multicentre international randomized controlled trial (CONCEPTT) the continuous glucose monitoring in pregnant women with T1DM showed strong correlation of TIR with better outcomes [24] The same group recently used rt-CGM and isCGM in 186 pregnant women to understand to what extent are CGM-derived measure of glucose control associated with large for gestational age infant (LGA) and neonatal outcome. Using either Dexcom G4 or Freestyle Libre CGM device Kristensen et al. calculated TIR, below or above pregnancy glucose target, CV%, SD of mean glucose, mean amplitude of glucose excursion (MAGE). They found no difference in maternal or neonatal outcomes between women using rt-CGM and isCGM and demonstrated that 5–7% lower TIR during the second and third trimesters was associated with increased risk of LGA and neonatal outcome, including macrosomia, shoulder dystocia, neonatal hypoglycemia. Interestingly, they support the non-inferior use of isCGM as technology ease of use, low cost, safe and accurate in pregnancy [24]. In a commentary of this study, Helen Murphy suggests that for optimal obstetric and neonatal outcomes, women should aim to reach a TIR > 70% and a time above range < 25%, as early as possible during pregnancy. Those who can’t achieve this target should be encouraged that any 5% increase in TIR is associated with clinically relevant improvements in neonatal health [25].”

Relationship between CGM-derived glycemic variables and the corresponding HbA1c levels were also found by analyzing individual-level data from four randomized clinical trials [27]. Those lasted ≥ 24 weeks, had end-of-study HbA1c levels and at least 2 weeks of continuous glucose monitoring data collected from 530 adults with T1DM and insulin-requiring T2DM. Participants were categorized based on end-of-study HbA1c levels ranging from < 6.5 to ≥ 8.5% and were separated into categories based on CGM-derived metrics. HbA1c was strongly correlated with mean glucose value (r = 0.80), TIR (r = − 0.75) and percentage of glucose values > 250 mg/dL (r = 0.729), but was weakly correlated with the percentage of glucose values < 70 mg/dL (r = − 0.39) or < 54 mg/dL (r = − 0.21). More than 90% of participants with either mean glucose < 140 mg/dL or time in range > 80% had HbA1c levels ≤ 7.0%. For participants with HbA1c ≥ 8.0%, the median TIR was 44%, with 90% of participants having a TIR < 59%.

TIR has also evaluated in the intensive care unit (ICU) scenario. Omar et al. [28] determined the whole time of insulin infusion (A) and the time within the proposed target range (B) during insulin infusion and expressed TIR as TIR = (B/A) × 100. They found that patients with more than 80% TIR, with or without diabetes, had better outcomes (wound infection, lengths of ventilation, and ICU stay) than those with less than 80% TIR. Additionally, they had less hypoglycemia. Krinsley and Preiser [29] had previously found that survival in critically ill patients without diabetes is strongly associated with TIR (70 to 140 mg/dL) above 80%. Their findings are independent of the ICU length of stay and severity of the individual’s illness. The authors suggest that individualized algorithms for patients with and without diabetes could replace published working guidelines that may be unnecessarily restrictive.

There are many methods described in the literature to evaluate glycemic control. Rodbard evaluated various metrics of glycemic control, and compared TIR, time in hypoglycemia (TBR) and Time in Hyperglycemia (TAR) with previously described risk indices, intending to validate metrics for quality of glycemic control, hypoglycemia and hyperglycemia [30]. The analysis of the mathematical properties of these methods were described in detail through linear regressions and correlations between conceptual groups. The report consisted of different “risk indices” of glycemic control (M100, Blood Glucose Risk Index, Glycemic Risk Assessment Diabetes Equation, Index of Glycemic Control, J-Index, Low Blood Glucose Index (LBGI), percentage of GRADE attributable to hypoglycemia (GRADE % Hypoglycemia), Hypoglycemia Index, High Blood Glucose Index (HBGI), percentage of GRADE attributable to hyperglycemia (GRADE %Hyperglycemia) and Hyperglycemia Index and suggested that it is unlikely that those risk indices could provide additional information. Of interest, TIR was highly negatively correlated with  %TAR but poorly correlated with  %TBR. Thus, for the SBD experts, TIR, TBR, and TAR are understandable and straightforward criteria with high correlation to other glycemic metrics that are more complex to calculate and more challenging to understand.

### Future directions

Usage of CGM enabled diabetologists, endocrinologists and diabetes educators to analyze more than one component of glycemic control. One of the possibilities for the future is the combination of various metrics trying to better define glycemic control [31].

Composite indices could have a numeric, visual or even having both indices aligned together. Composite indices that have a numeric representation include the Index of Glycemic Control (IGC), Q-score, Composite Continuous Glucose Monitoring Index (COGI) and others. Moreover, those with a visual representation include the graphical display of CGM [32, 33], and indices with both a numeric and visual representation include the Hypo-Triad and the Comprehensive Glucose Pentagon (CGP) [34].

The Q-Score is a new metric suitable to screen for CGM profiles that require therapeutic action. It identified five primary factors that determined CGM profiles (central tendency, hyperglycemia, hypoglycemia, intra- and inter-daily variations) where one parameter from each factor was selected for constructing the formula. The Q-Score should allow the categorization of glycemic control from very good to poor it also allows identification of factor(s) underlying the profiles that are mainly responsible for the quality of metabolic control in each patient [35].

Leelarathna et al. created the COGI which consists of three key components of glucose control, as assessed by CGM: TIR, TBR, and GV. It was evaluated in adults with T1DM, using hybrid closed-loop (HCL) therapy and MDI therapy combined with rtCGM [36]. They weighted each of the components differently, determined arbitrarily by their importance in 50% for TIR, 35% for TBR and 15% for GV. COGI ranges from 0 to 100; a one percent increase of time < 70 mg/dL is equivalent to almost 5% reduction of TIR while 9 mg/dL increase in SD is equivalent to 3% reduction in TIR. They found that patients in CSII with HbA1c between 7.5 and 10%, COGI was significantly higher in HCL compared to sensor-augmented pump therapy, mean (SD) 60.3 (8.6) versus 69.5 (6.9), (p < 0001), and those CSII users with HbA1c < 7.5% COGI improved from 59.9 (11.2) to 74.8 (6.6) (p < 0001). MDI users had similar results. The authors concluded that COGI is a concise metric that unifies three important aspects of CGM data and it could be used to evaluate glucose control and to demonstrate the differences between different treatment modalities.

Finally, the CGP which includes five key metrics of glycemic control derived from CGM such as mean sensor glucose, GV, severity of hypo- and hyperglycemia, and time out of range (the inverse of TIR), but eliminates HbA1C, demonstrates glycemic control both numerically and visually [31]. It showed potential to enable health care providers, investigators and patients to better understand the components of glycemic control and the effect of several interventions on the individual elements of that control. This can be done on a daily, weekly, or monthly basis [31, 34].

Gathering more than one metric is an attractive idea when analyzing diabetes control and might be used more frequently over the next years. Currently, however, we have no strong evidence about composite indexes, so in our opinion, we should get used to the concept of TIR before moving to other combined metrics.

The big challenge is to implement this practice more widely in countries with a limited health public and private budget as it occurs in Brazil. Could CGM be used intermittently, for example, 14 days every 3 months, looking for patterns of GV, TIR, and percentage of hyper and hypoglycemia, before the medical appointments? “It is important to note that there are differences between the professional short-term blinded continuous glucose monitoring (pCGM) and the personal CGM (real-time- rtCGM). Personal rtCGM allows an individual to self-monitor how his blood glucose responds to various lifestyle factors on a daily basis, while pCGM is masked to the user at the time of wear. Some studies evidenced improvement in HbA1c with the blinded device in T1D and T2D patients, others did not show any difference regarding metabolic control [37–39]. In a 3-day blinded CGM study using the iPRO device in 106 consecutive individuals, the authors concluded that the procedure was ineffective for improving HbA1c levels in adults with type 1 and 2 diabetes [38]. Nevertheless, the real effect of the pCGM is still controversial and need more evidence, as the studies were conducted in a small number of subjects and heterogeneous populations, with limited data in T1DM children < 7 years and no cost-effectiveness evaluation.”

Alternatively, CGM could be indicated for those on multiple doses of insulin analogues who still have severe hypo or nocturnal hypoglycemia, before witching to CSII? Should physicians prescribe a sensor-augmented pump for all young children and for those who already use CSII and persist with nocturnal or severe hypoglycemia? Is the seven-point SMBG enough for TIR determination or periodic use of CGM is essential in the clinical practice? Clinical trials are urgently needed to elucidate these questions and establish adequate cost-effective clinical guidelines for middle-income countries.

It is critical to emphasize that it has already been proven, even in developing countries, that increase in number of scans/days is related to increase in TIR and reduction in time in hypo and eA1c. These results suggested that better glucose control can be achieved with sensors, independently of other possible confounding factors. Although the seven-point SMBG has been used for TIR evaluation, there is recent evidence that results with this method might significantly differ from those obtained through CGM [40], with overestimation of % of hypo and hyperglycemia.

## Conclusion

After reviewing the available data, the Brazilian Diabetes Society recommends the use of TIR as a new and very useful tool to evaluate glycemic control. Data should be extracted from sensors, for at least 10 days, but preferentially for 14 days. In the absence of sensors, more studies should be done to validate SMBG obtained at different times of the day and with the amount of data high enough to simulate the time in specific ranges.

Nevertheless, the most comprehensive data available until now are in T1D, they are considered for T2D as well.

Strategies to implement the use of this new metric into medical practice in Brazil and other developing countries middle-income countries is still a challenge. Cost-effective studies are needed to help understand the possibility of having sensors and include TIR evaluation in clinical practice nationwide.

## Data Availability

Not applicable.

## Abbreviations

ADA: American Diabetes Association
BG: Blood glucose
CIMT: Carotid intima: media thickness
CGM: Continuous glucose monitoring
COGI: Composite continuous glucose monitoring index
CSII: Continuous subcutaneous insulin infusion
DCCT: Diabetes Control and Complications Trial
eA1c: estimated HbA1c
EASD: European Association for the Study of Diabetes
GV: Glucose variability
HBGI: High Blood Glucose Index
HCL: hybrid closed: loop
IC-TIR: The International Consensus in Time in Range
ICU: Intensive care unit
IHSG: International Hypoglycaemia Study Group
isCGM: Intermittent scan CGM
LBGI: Low Blood Glucose Index
MARD: Mean absolute relative difference
MDI: Multiple daily injections
RBC: Red blood cell
rt- CGM: Real-time CGM
SBD: Brazilian Society of Diabetes
SMBG: Self- monitoring blood glucose
TIR: Time in Range
TAR: Time Above Range
TBR: Time Bellow Range
T1DM: Type 1
T2DM: Type 2
